# Metal-Based Graphical SiO_2_/Ag/ZnS/Ag Hetero-Structure for Visible-Infrared Compatible Camouflage

**DOI:** 10.3390/ma11091594

**Published:** 2018-09-03

**Authors:** Dong Qi, Xian Wang, Fu Chen, Yongzhi Cheng, Rongzhou Gong

**Affiliations:** 1 School of Optical and Electronic Information, Huazhong University of Science and Technology, Wuhan 430074, China; qidong@hust.edu.cn (D.Q.); zs101141@163.com (F.C.); rzhgong@hust.edu.cn (R.G.); 2 School of Information Science and Engineering, Wuhan University of Science and Technology, Wuhan 430081, China; chengyz@wust.edu.cn

**Keywords:** metal-based structure color, ultra-low infrared emissivity, compatible camouflage, skin depth, multilayer structure

## Abstract

A brand-new approach to realizing visible-infrared compatible camouflage is proposed based on a metal-based graphical hetero-structure (MGHS) SiO_2_/Ag/ZnS/Ag. For different thicknesses (20, 40, and 60 nm) of color-controlling sub-layer, high-contract and large-span structure colors (yellow, navy, and cyan) were observed due to reintroducing constructive interference with a matching intensity of reflected waves. Ultra-low infrared emissivity values of 0.04, 0.05, and 0.04 (with high average reflectance values of 95.46%, 95.31%, and 95.09%) were obtained at 3–14 μm. In addition, the well-performing trisecting-circle structure further indicates that it is feasible to design on-demand compatible camouflage patterns using the easily-prepared MGHS.

## 1. Introduction

With the rapid development of modern detection industries, traditional single stealth approaches, such as visible-light camouflage [1,2] and infrared emissivity engineering [3,4,5,6], are severely threatened by dual- and multi-approach target detectors [7,8], which were proposed and practically applied in recent years. Hence, the impending compatible camouflage demand of visible light and infrared has aroused extensive research interests, such as developing colored photonic crystals [9] and chemical pigments [10,11]. Nevertheless, complicated technology and non-controllable colors are still direct obstacles for the large-scale application of camouflage combined with various structure colors and ultra-low emissivity (*ε*). In order to overcome this limitation, extremely thin metallic film, as a potential candidate, has been proposed for infrared camouflage due to its being easily prepared and ultra-low *ε* in whole infrared atmospheric windows [12,13]. Thus, the metal-based multi-layer films is a suitable candidate due to its combination of diversified interference colors (dielectric film) and ultra-low *ε* (metal film). However, up to now, little research has been performed to realize visible-infrared compatible camouflage using this design strategy.

**Figure 1:**
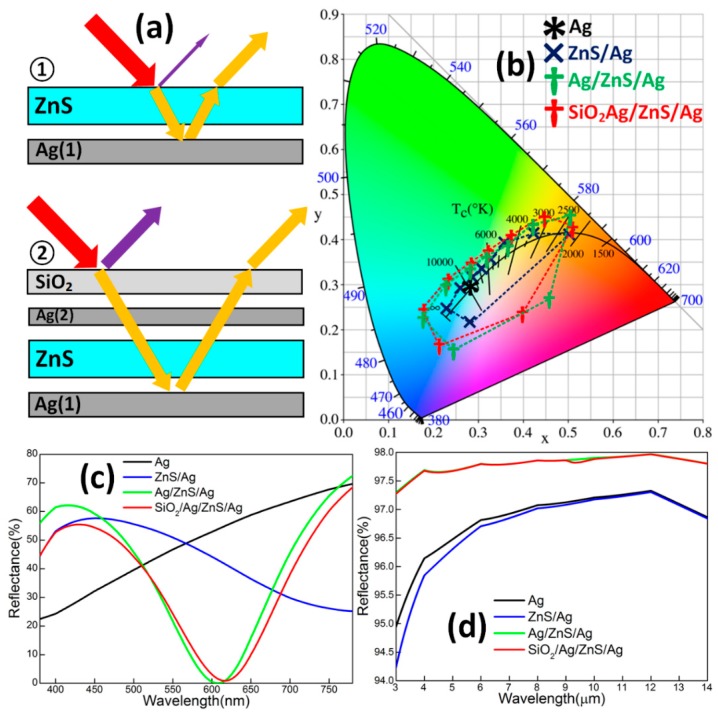
(**a**) The contrasting schematic illustrations of reflections between Ag/ZnS and the designed SiO_2_/Ag/ZnS/Ag. (**b**) The simulated chromaticity coordinates of different structures from 10 nm to 90 nm (interval: 10 nm). The reflection spectrum of different structures at (**c**) visible-light 380–780 nm and (**d**) infrared 3–14 μm corresponding to *d*_ZnS_ = 60 nm.

In this work, a simplified composite hetero-structure SiO_2_/Ag/ZnS/Ag was firstly proposed to make breakthroughs in the following three aspects: simplifying the process, reintroducing matching constructive interference for metallic film, and increasing infrared properties (Figure 1a). From the bottom to the top of Figure 1a, the following steps are shown. An infrared-reflection sub-layer (Ag: 15 nm) was deposited on a quartz substrate to serve as the basic structure. The color-controlling sub-layer (ZnS: 20, 40, or 60 nm) was subsequently deposited, mainly to realize a high-contract structure color based on constructive interference. In order to illustrate the functions of each sub-layer, variable *d*_ZnS_ was provisionally set as 60 nm in Figure 1b–d. Ag 10 nm in thickness was designed as a reflection-enhancing sub-layer. It plays a crucial role in providing the matching intensity of reflected waves of both metal interfaces shown in Figure 1a,b. This means reintroducing obvious peaks and troughs of the visible-light reflection spectrum. This function is also verified by larger chromaticity coordinate spans (SiO_2_/Ag/ZnS/Ag, red) compared with the one (Ag/ZnS, blue) in the calculated chromaticity diagram (Figure 1c) for various ZnS thicknesses (from 10 to 90 nm, at intervals of 10 nm). This sub-layer can improve infrared reflectance simultaneously (Figure 1d). The top-covered sub-layer (SiO_2_: 20 nm) is employed to protect the functional structure from oxidation and corrosion, which has a negligible effect on spectral properties (Figure 1b–d). In addition, good performance samples with a graphical “trisecting-circle” hetero-structure were prepared to further demonstrate the extendibility of the designed metal-based graphical hetero-structure (MGHS) SiO_2_/Ag/ZnS/Ag in deformation and digital camouflage [14,15].

## 2. Experimental Design

The designed composite film SiO_2_/Ag/ZnS/Ag was deposited on optical glass K9 (diameter *φ* = 50 mm, thickness *d* = 3 mm). The thickness of each sub-layer is listed as follows: *d*_SiO2_ = 20 nm; *d*_Ag(1)_ = 15 nm; *d*_Ag(2)_ = 10 nm; and *d*_ZnS_ = 20, 40, and 60 nm. For non-absorbent materials (k ≈ 0), the average refractive index *n*_ZnS_ = 2.35 and *n*_SiO2_ = 1.46 can be given for facilitating the calculation due to small shift in the whole designed waveband [16,17]. Nevertheless, the dispersion relationship of Ag cannot be neglected, with refraction index and extinction coefficient are fitted as follows [18].

(1) $$n=9\times 10^{-8}\lambda^{2}+3\times 10^{-4}\lambda -0.1631$$

(2) $$k=7\times 10^{-8}\lambda^{2}+7.4\times 10^{-3}\lambda -0.8432$$

**Figure 2:**
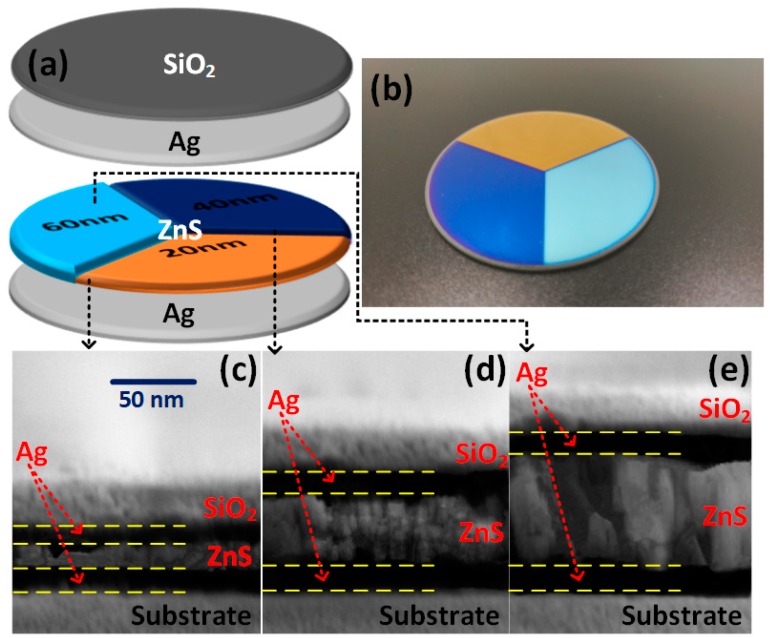
(**a**) The structural schematic diagram of MGHS. (**b**) The photograph of the actual MGHS sample. The cross-section micro-morphology of the designed SiO_2_/Ag/ZnS/Ag with obviously alternating and proportional sub-layers for *d*_ZnS_ = (**c**) 20, (**d**) 40, and (**e**) 60 nm.

SiO_2_ (purity: 99.99%), Ag (purity: 99.99%), and ZnS (purity: 99.99%) pellets were prepared for electron beam evaporation (EBE, Rankuum ZZS1100-8/G) with ion-beam assisted deposition (IBAD, Ar flow rates: 7 sccm). The corresponding deposition rates were approximately steady at 6, 10, and 3 Å/s, respectively, which were monitored by a quartz crystal monitor (Inficon IC6, Bad Ragaz, St. Gallen, Switzerland). The base pressure during deposition was maintained at 1 × 10^−3^ Pa, without external substrate heating. Figure 2a shows the schematic illustration of MGHS. 120° and 240° sectorial masks were machined and successively bound to deposit ZnS 20 nm in thickness on ZnS (20 nm)/Ag substrate.

## 3. Results and Discussion

The actual MGHS specimens, trisecting-circle structures, are presented in Figure 2b. Their profile and coloration are in good accordance with the original design. Three structure colors, yellow, navy, and cyan, have been intuitively observed. Cross-section photographs with proportional bright (dielectric) and dark (metal) strips obtained by field emission scanning electron microscope (FESEM, FEI Sirion 200, FEI, Hillsboro, OR, USA) (Figure 2c–e) demonstrate the successful preparation and structural variation of MGHS. The measurement conditions were extra high tension (2.00 kV), magnification (104.55 KX), and working distance (4.9 mm). According to the measuring scale, there exists an inevitable and small relative deviation *δ*_T_ of 3.08%, 4.71%, and 4.76% between the actual total thickness *d*_T_ of approximately 67, 89, and 110 nm and the original values 65, 85, and 105 nm, respectively. This is primarily caused by imperfect preparation and measurement technique. For example, tooling factors 110%, 100%, and 114% of SiO_2_, Ag, and ZnS were the approximate adjustment values for our specific equipment according to the formula $Tooling\%=TF_{i}\times (T_{m}/T_{x})$, where *TF*_i_, *T*_m,_ and *T*_x_ are initial tooling factor, actual thickness, and thickness at the crystal (IC6), respectively [19]. In brief, except for tiny uneven sections, macro- and micro-structure measurements provide convictive proofs for the complete fabrication of designed MGHS.

**Figure 3:**
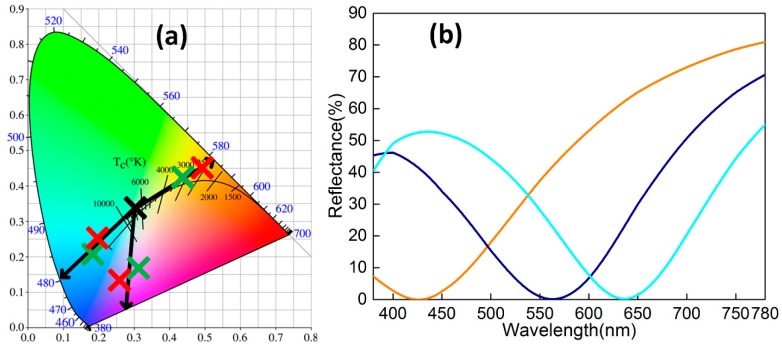
(**a**) The simulated (green crosses) and measured (red crosses) CCs with similar color features in chromaticity diagram. (**b**) The experiments measured a reflection spectrum for the 380–780 nm range for *d*_ZnS_ = 20, 40, and 60 nm.

Regarding visible-light color rendering, the introduction of the reflection-enhancing Ag sub-layer provides a matching intensity of constructive interference waves and enriches the color variance. Combining a skin depth [20] of Ag at δ=λ/4πk with its *k* − *λ* relation (Equation (2)), the nonlinear fitting formula can be expressed as $\delta \approx 31.629\times \lambda^{-0.135}$ (nm) which is monotone and decreasing throughout the whole range of 380–780 nm. Ag 10 nm in thickness was employed as a reflection-enhancing sub-layer due to the numerical relationship $10<\delta_{780}(\approx 12.3)$ nm, which indcicates the coexistence of reflection and transmission. As shown in Figure 3b, three reflection spectra have been measured (Shimadzu UV-3600Plus, Shimadzu, Nakagyo-ku, Kyoto, Japan) in the 380–780 nm range, which corresponds to pre-designed sector areas of yellow, navy, and cyan with various thicknesses (*d*_ZnS_ = 20, 40, and 60 nm). This model of the spectrophotometer possesses three detectors (Photomultiplier Tube, InGaAs, PbS, Shimadzu, Nakagyo-ku, Kyoto, Japan) with a high sensitivity of 0.00003 Abs and resolution of 0.1 nm. According to the following formulas from the CIE 1931 standard [21], the correlation between reflection spectrum *R*(*λ*) and chromaticity coordinates CCs (*x*, *y*) is established by tristimulus values (*X*, *Y*, *Z*),

(3) $$M=100\frac{\int_{\lambda }S(\lambda )R(\lambda )\bar{m}(\lambda )d\lambda }{\int_{\lambda }S(\lambda )\bar{y}(\lambda )d\lambda }$$

(4) $$\left(x,y)=(\frac{X}{X+Y+Z},\frac{Y}{X+Y+Z}\right)$$

where *M* and *m* indicate *X*, *Y*, *Z* and *x*, *y*, *z*. *S*(*λ*), pre-defined by chromatics, is the relative spectral power distribution of the illuminant D65. Therefore, as depicted in Figure 3a, corresponding actual CCs (Colorimeter DC-P3) are measured as (0.4927, 0.4511), (0.2581, 0.1354), and (0.1973, 0.2564). For *d*_ZnS_ = 20 and 60 nm, the CCs from the experiment approximatively share the uniform dominant wavelengths (collinear) *λ*_d_ = 582 and 481 nm with the simulation data (0.4342, 0.4293) and (0.1802, 0.2112), This definitely demonstrates color consistency. For *d*_ZnS_ = 40 nm, a similar color feature is obtained, but there exists no practical *λ*_d_ because the intersection of the spectral locus and fitting line is on the “purple line”. Thickness-sensitive color changing under the purple-blue area (Figure 1b) in a chromaticity diagram causes the accuracy of fabrication tolerances to be hard to control. This simultaneously produces a small deviation of CCs. Thus, as expected, a certain MGHS design exhibits strong expansibility in visible-light background adaptation.

**Figure 4:**
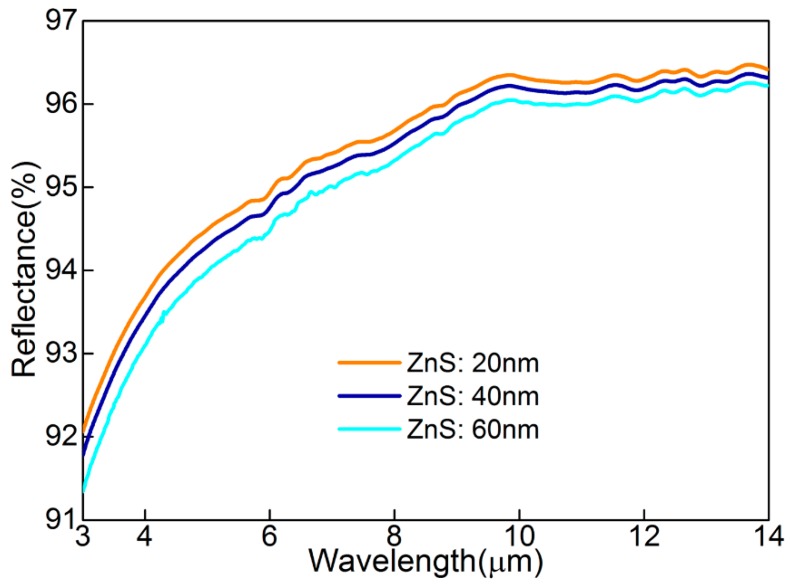
The experiment measured infrared reflection spectrum for the 3–14 μm range for *d*_ZnS_ = 20, 40, and 60 nm.

Similar to visible-light, the infrared spectral characteristic of MGHS also depends on the thickness (*d*) and extinction coefficient (*k*) of Ag film, which are both considered in refitting skin depth, $\delta =\lambda /4\pi k\approx 6\times 10^{-9}\lambda^{2}-2\times 10^{-4}\lambda +11.401$ (nm), for 3–14 μm. A maximum $\delta_{3\mu m}\approx 10.9(<(10+15))$ nm makes the designed Ag film an excellent candidate for infrared high reflectance (*R*) (i.e., low absorption and negligible transmission). On the other hand, the theoretical infrared emissivity of $\varepsilon =4n/({\left(n+1\right)}^{2}+k^{2})$ [22] indicates that an ultra-low *ε* can be quantifiably achieved from Equations (1) and (2). Figure 4 shows infrared reflection spectrum (3–14 μm) of three sectors, which were measured by Fourier transform infrared spectroscopy (PerkinElmer Frontier, manufacturer, Waltham, MA, USA). This spectrometer possesses a unique detector (Deuterated Triglycine Sulfate, DTGS) with a high resolution of 0.3 nm. As for different ZnS thicknesses (*d*_ZnS_ = 20, 40, and 60 nm), it can be found that there was an ultra-high average reflectance (i.e., $\bar{R}$ = 95.46, 95.31%, and 95.09%, respectively), with a small deviation mainly caused by varying *d*_ZnS_. The incremental tendency of *R* towards a long-wave is generated by the decreasing *δ* and increasing *k*. In addition, the room-temperature surface infrared emissivity (Surface Optics Corporation, SOC-410 DHR, San Diego, CA, USA) of MGHS has been verified as *ε* = 0.04, 0.05, and 0.04. The correctness of these values are indirectly validated by Kirchhoff’s law [9], $\varepsilon \approx 1-\bar{R}$, for the non-transparency in the 3–14 μm wavelengths.

## 4. Conclusions

In summary, a brand-new compatible camouflage strategy of visible-infrared, utilizing MGHS SiO_2_/Ag/ZnS/Ag, has been proposed and successfully prepared. By comparing actual thickness with skin depth (*δ*), different functions of MGHS in visible-light and infrared have been quantifiably illuminated. As a consequence, due to the reintroduction of matching waves of constructive interference, three colors—yellow, navy, and cyan—on the trisecting-circle specimens were clearly observed corresponding to different *d*_ZnS_ (of 20, 40, and 60 nm). In addition, approximately equal ultra-low infrared emissivity values of 0.04, 0.05, and 0.04, respectively, were obtained at 3–14 μm. We therefore believe that the designed MGHS will provide an important reference for on-demand visible-infrared compatible camouflage.

## References

[1] Morin S.A., Shepherd R.F., Kwok S.W., Stokes A.A., Nemiroski A., Whitesides G.M. (2012). Camouflage and display for soft machines. Science.

[2] Diao Z., Kraus M., Brunner R., Dirks J.H., Spatz J.P. (2016). Nanostructured stealth surfaces for visible and near-infrared light. Nano Lett..

[3] Chu H.T., Zhang Z.C., Liu Y.J., Leng J.S. (2016). Silver particles modified carbon nanotube paper/glassfiber reinforced polymer composite material for high temperature infrared stealth camouflage. Carbon.

[4] Liu X.F., Lai Y.K., Huang J.Y., Al-Deyab S.S., Zhang K.Q. (2015). Hierarchical SiO_2_@Bi_2_O_3_ core/shell electrospun fibers for infrared stealth camouflage. J. Mater. Chem. C.

[5] Phan L., Walkup W.G., Ordinario D.D., Karshalev E., Jocson J.M., Burke A.M., Gorodetsky A.A. (2013). Reconfigurable infrared camouflage coatings from a cephalopod protein. Adv. Mater..

[6] Ye X.Y., Zheng C., Xiao X.P., Cai S.G. (2015). Synthesis, characterization and infrared emissivity study of SiO_2_/Ag/TiO_2_ “sandwich” core-shell composites. Mater. Lett..

[7] Green R.O., Eastwood M.L., Sarture C.M., Chrien T.G., Aronsson M., Chippendale B.J., Faust J.A., Pavri B.E., Chovit C.J., Solis M. (1998). Imaging spectroscopy and the airborne visible/infrared imaging spectrometer (AVIRIS). Remote Sens. Environ..

[8] Verevkin A., Zhang J., Sobolewski R. (2002). Detection efficiency of large-active-area NbN single-photon superconducting detectors in the ultraviolet to near-infrared range. Appl. Phys. Lett..

[9] Zhao Y.Q., Zhao Y., Hu S., Lv J.T., Ying Y., Gervinskas G., Si G. (2017). Artificial structural color pixels: A review. Materials.

[10] Yuan L., Hu J., Weng X.L., Zhang Q.Y., Deng L.J. (2016). Galvanic displacement synthesis of Al/Ni core–shell pigments and their low infrared emissivity application. J. Alloys Compd..

[11] Wang K.Z., Wang C.X., Yin Y.J., Chen K.L. (2017). Modification of Al pigment with graphene for infrared/visual stealth compatible fabric coating. J. Alloys Compd..

[12] Xu J.J., Tang J.F. (1989). Optical properties of extremely thin films: Studies using ATR techniques. Appl. Opt..

[13] Smith G.B., Niklasson G.A., Svensson J.S.E.M., Granqvist C.G. (1986). Noble-metal-based transparent infrared reflectors: Experiments and theoretical analyses for very thin gold films. J. Appl. Phys..

[14] Wang J.T., Xu W.D., Qu Y., Cui G.Z. (2016). Research on measurement method of optical camouflage effect of moving object. Optical Measurement Technology and Instrumentation. Int. Soc. Opt. Photonics.

[15] Xue F., Xu S., Luo Y.T., Jia W. (2016). Design of digital camouflage by recursive overlapping of pattern templates. Neurocomputing.

[16] Debenham M. (1984). Refractive indices of zinc sulfide in the 0.405–13 μm wavelength range. Appl. Opt..

[17] Gao L.H., Lemarchand F., Lequime M. (2012). Exploitation of multiple incidences spectrometric measurements for thin film reverse engineering. Opt. Express.

[18] Ciesielski A., Skowronski L., Trzcinski M., Szoplik T. (2017). Controlling the optical parameters of self-assembled silver films with wetting layers and annealing. Appl. Surf. Sci..

[19] Riley B.J., Sundaram S.K., Johnson B.R., Saraf L.V. (2006). Summary of Chalcogenide Glass Processing: Wet-Etching and Photolithography.

[20] Scalora M., Bloemer M.J., Pethel A.S., Dowling J.P., Bowden C.M., Manka A.S. (1998). Transparent, metallo-dielectric, one-dimensional, photonic band-gap structures. J. Appl. Phys..

[21] Wyszecki G., Stiles W.S. (1982). Color Science: Concepts and Methods, Quantitative Data and Formulae.

[22] Barrat S., Pigeat P., Dieguez I., Bauer-Grosse E., Weber B. (1995). Observation of spectral and normal emissivity as a method of surface control during the growth of diamond films deposited by a microwave plasma-assisted CVD technique. Thin Solid Films.

